# Modelling the Potential Human Exposure to Japanese Encephalitis Virus (JEV) in Case of Introduction into Reunion Island

**DOI:** 10.1155/2023/3118640

**Published:** 2023-06-20

**Authors:** Héléna Ladreyt, Claire Garros, Nausicaa Habchi-Hanriot, Marlène Dupraz, Thierry Baldet, Véronique Chevalier, Benoit Durand

**Affiliations:** ^1^UMR ASTRE, CIRAD, INRAE, University of Montpellier, Montpellier, France; ^2^ARS-La Réunion, Sainte-Clotilde, France; ^3^UMR ASTRE, CIRAD, Sainte-Clotilde, La Réunion, France; ^4^UMR ASTRE, CIRAD, Antananarivo, Madagascar; ^5^Epidemiology and Clinical Research Unit, Institut Pasteur de Madagascar, Antananarivo, Madagascar; ^6^Epidemiology Unit, Laboratory for Animal Health, ANSES, University Paris Est, Maisons-Alfort, France

## Abstract

Japanese encephalitis virus (JEV) is a vector-borne zoonotic virus and the leading cause of human acute encephalitis in Asia. Continuous human and commercial exchanges between Southeast Asia where JE is endemic and Reunion Island increase the risk of introducing JEV on the island, where putative vectors of JEV such as *Culex quinquefasciatus* and amplifying hosts such as pigs are present. Each of the 255 Reunionese pig farms was assumed to harbor a *Cx. quinquefasciatus* population and, together with the competent hosts: pigs and poultry and noncompetent hosts: humans, dogs, and cattle, located within a radius of 1 km, formed an epidemiological unit. We used a deterministic compartmental model to investigate whether these epidemiological units could be invaded by JEV in the event of an introduction. Since the vector population size changes seasonally, we computed the basic reproduction number (*R*_0_) using vector population sizes ranging from 100 to 100,000 vectors for each of the 255 epidemiological units. The size of the potentially exposed human population was calculated in the case where the virus would be introduced in a single epidemiological unit and in the extreme case where the virus would have spread over the whole island. For a vector population of 1,000 vectors per unit, 2 out of 255 units had an *R*_0_ ≥ 1. With 50,000 vectors per unit, more than 75% (193/255) of the units had an estimated *R*_0_ ≥ 1, representing a median of approximately 2,500 potentially exposed people if JEV was introduced in a single unit, and about 140,000 potentially exposed people if JEV had expanded throughout the island. The unit located a few kilometers from the large port area of Reunion Island had an estimated *R*_0_ ≥ 1 with at least 10,000 vectors, making it a potential gateway to JEV given a virus introduction of infected vectors.

## 1. Introduction

Anthropogenic landscape changes, particularly habitat destruction and fragmentation, encroachment on wilderness areas, and global warming, contribute to the expansion of mosquito vectors and the emergence of arboviruses [1–6]. Human and trade exchanges contribute to changes in the distribution and intensity of arbovirus transmission by impacting vector and host distribution [5, 7–12] and have already led to the emergence of arboviruses in new regions such as West Nile fever virus in the United States, and Zika virus in Brazil [13, 14].

Reunion Island (2,512 km^2^, 863,100 inhabitants in 2020) is a French overseas department located in the Indian Ocean, 700 km east of Madagascar [15]. Some vector-borne diseases have already been introduced in Reunion Island and have caused or are causing serious public health problems. Two human arboviruses transmitted in the island by *Aedes albopictus* are particularly noteworthy: chikungunya virus, likely introduced from East Africa via the Comoros Islands, lead to an epidemic affecting nearly 40% of the population in 2005-2006 [16] and dengue virus with a first documented epidemic in the island in 1977-1978 followed by sporadic epidemics in the 2000s [17]. Since 2018, dengue seems to have become endemic in the island with (i) an uninterrupted circulation of the virus including during the austral winter, (ii) a cocirculation of different serotypes, and (iii) an increase in the number of cases with severe forms and deaths [18]. Vector-borne zoonotic diseases have recently emerged in Reunion Island, such as flea-borne murine typhus [19]. Two vector-borne zoonotic viruses are under special surveillance because they circulate in the region: Rift Valley fever virus (circulating in Madagascar [20] and in Mayotte [21]) and West Nile fever virus, circulating in Madagascar and for which antibodies have been detected in horses in Reunion Island, with no observed indigenous human cases [22, 23].

Japanese encephalitis virus (JEV) is the major cause of human acute viral encephalitis in Asia, accounting for approximately 100,000 cases and over 25,000 deaths in 2015 [24]. Despite the implementation of vaccination programs in some countries [25], Japanese encephalitis (JE) remains a significant public health issue with fatality rates as high as 30% and severe neurological sequelae in 30–50% of survivors [26–30]. Japanese encephalitis is considered an emerging zoonotic disease and is widely distributed across Southeast Asia. In 2016, a locally transmitted case of JE was reported in Angola [31], and the disease reemerged in Australia with large detection in pigs and a substantial number of locally acquired human cases in early 2022 [32].

Transmission of JEV is generally assumed to occur from Ardeid birds (wild reservoir hosts) or domestic pigs (main amplifying hosts [33]) to human through the bites of *Culex* spp. and possibly some *Aedes* spp. mosquitoes [34]. However, recent laboratory studies suggest that domestic birds are competent hosts for JEV [35] and could play a significant role in JEV circulation in pig-free endemic areas [36]. Like humans, cattle and dogs are noncompetent hosts for JEV but can be exposed to it by being bitten by opportunistic vectors carrying JEV [37–39].

Twelve species of mosquitoes (belonging to five genera: *Aedes*, *Anopheles*, *Culex*, *Lutzia,* and *Orthopodomyia*) are currently found on Reunion Island [40]. Among them, *Ae. albopictus* and *Cx. quinquefasciatus* are the most abundant and are commonly found in urban, periurban, and rural areas; sometimes up to 1,200–1,400 m of altitude [40]. *Culex quinquefasciatus* is a vector of JEV in Asia [41] and occurs in both urban and rural areas given the high diversity of suitable breeding sites, such as polluted, organic-rich, and clear waters [40]. Females are considered opportunistic feeders, obtaining blood meals from humans, domestic mammals, and birds [40]. Originating from Asia, *Cx. tritaeniorhynchus* is the main vector of JEV in Asia but is also present throughout the entire coastline of Reunion island [40]. However, populations of *Cx. tritaeniorhynchus* in Reunion Island are not very abundant and are usually restricted to areas surrounding ponds and wetlands. The preferred larval sites of *Cx. tritaeniorhynchus* are often natural areas that receive a lot of sunlight, such as rock outcroppings and flooded meadows [40]. It is also an opportunistic feeder; preferentially biting cattle and pigs, but will occasionally bite humans when its preferred hosts are missing [39].

Pig farming represents the second largest animal production on Reunion Island, with 255 farms recorded in 2019 [42]. Broiler production in Reunion Island, on the other hand, is used as diversification production and is characterized by the small size of its farms (reference farm at 600 m^2^) and the high number of smallholders. The accessible and habitable territory in Reunion Island being restricted due to the landscape, farms are concentrated in coastal and intermediate areas, up to about 900 meters of altitude, corresponding to urban and periurban areas where human population and mosquito densities are the highest [43–45]. 

In the Indian Ocean, trade is important and growing. Numerous commercial cargo lines exist between Reunion Island and JEV endemic regions such as Southeast Asia, ensuring the importation of goods in 18–30 days [46, 47], which may be consistent with the survival of infected *Culex* spp. on board, living on average 25 days [48].

JEV circulates mainly in tropical climates like Cambodia. For example, Phnom Penh averages an annual temperature of 27.8°C (minimum 23.8°C and maximum 32.5°C on average) and a rainfall of 1,635.6 mm per year [49]. Climate in Reunion Island is tropical with hot and rainy summers (November–April) and cool and dry winters (May–October). On the periphery of the island, i.e., in the low and medium altitude areas where pig farms are located, the annual temperature averages 22–34°C (minimum 18–24°C and maximum 24–34°C) and the annual rainfall averages from 500 to 1,500 mm in the west and from 1,500 to 4,000 mm in the east, making the island a tropical climate zone [50].

Due to trade links with Southeast Asia, the presence of potential JEV vectors and competent hosts, and a tropical climate, JEV could be introduced to and spread in Reunion Island. The objective of this study was to analyze the ability of epidemiological units composed of pigs, poultry, cattle, dogs, humans, and vectors in Reunion Island to allow JEV to invade the units in the event of an introduction and to estimate the size of the human population that would be exposed should it occur. To achieve this goal, we used an existing deterministic dynamic transmission model that incorporated pigs and poultry as competent hosts and cattle, humans, and dogs, as noncompetent hosts as well as *Cx. quinquefasciatus* mosquitoes as potential vectors of JEV. This model allowed reproducing serological field data in Cambodia, a disease-endemic region [36]. The basic reproduction number *R*_0_ is the expected number of secondary cases generated by a primary case in an entirely susceptible population [48]. *R*_0_ is the indicator commonly used to measure whether (*R*_0_ ≥ 1) or not (*R*_0_ < 1) a pathogen can invade a population [51, 52]. We used this model to estimate *R*_0_ in the epidemiological units, and calculated the size of the human population living in the units where the estimated *R*_0_ was greater than 1 and so that would be potentially exposed to JEV, first if the virus was introduced in a single epidemiological unit, and second if it had spread to the whole island.

## 2. Materials and Methods

### 2.1. Epidemiological Units

The larvae of the most abundant *Culex* spp. mosquito in Reunion Island, *Cx. quinquefasciatus*, pullulate in all urban waters rich in organic matter or polluted by detergents, as well as in rural areas in polluted waters of agri-food discharges, domestic water supplies and manure pits ([40], field observations). Wethus considered that each identified pig farm had at least one mosquito breeding site, namely the manure pit, which was therefore productive throughout the year.

*Culex tritaeniorhynchus* may be locally present in Reunion Island but is less abundant and more concentrated on the coastline, preferring natural clear water for its breeding sites [40]: the *Culex* spp. population on pig farms was assumed to be dominated by *Cx. quinquefasciatus*.

In Reunion Island, the 255 pig farms are mostly family-run, small in size, and in buildings open to the outside world, therefore not impervious to vectors. There are also a few open-air farms [42, 53]. Open-air poultry farming is widespread, with many family farms of the “backyard” type. The more industrial poultry farms vary greatly in size, ranging from 600 to about 28,000 places in buildings. Nevertheless, these buildings are open to the exterior, notably through ventilation systems [43, 53].

Regarding the noncompetent hosts in addition to humans, cattle are raised in extensive outdoor farms spread all over the island [54, 55], and domestic but also stray dogs are numerous and also live outdoors [56].

The modelled epidemiological units were then composed of the pig farms, the associated *Cx. quinquefasciatus* population, and competent (pigs from other farms, poultry) and noncompetent (humans, cattle and dogs) hosts that live within *Cx. quinquefasciatus* average flying distance (1 km) [48].

### 2.2. Data Sources

The sizes and locations of pig (*N* = 255 farms), poultry (*N* = 350 farms), and cattle (*N* = 1261 farms) farms were obtained from the Reunion Island sanitary protection association (GDS Réunion), Avi-pôle poultry cooperative, the French agricultural research and cooperation organization working for the sustainable development of tropical and Mediterranean regions (CIRAD) and veterinary services (DAAF Réunion, “SIGAL” database). The processing of the raw data and a georeferencing of the farms for which the exact location was unknown allowed us to obtain the geographical coordinates and the number of animals for each farm. The declarations of classified installations publicly available on the DAAF website of Reunion Island were used to complete the information on the number of animals, when this was missing. All data were anonymized.

Public census data of the French population (INSEE) were used for human spatial density (human population per km^2^) [44]. A ratio of humans to domestic dogs (1 : 3.8) estimated by a local official survey [56] was applied to the human spatial density to estimate the spatial density of domestic dogs on the island. This same survey estimated from field observations the density of stray dogs by INSEE territorial division (IRIS zones, *N* = 230), for which we obtained the data and GIS layers.

### 2.3. Model

The model was a deterministic compartmental susceptible-exposed-infective-recovered (SEIR) model, operating in continuous time, initially developed to simulate the transmission of JEV between *Culex spp*. and competent hosts (pigs, chickens, and ducks) and from *Culex spp*. to noncompetent hosts (cattle, dogs, and humans) in rural multihost systems in Cambodia. A detailed description of the model is given in Ladreyt et al. [36].

The average life expectancies of domestic animals, depending on the production systems, and of humans (1/*μ*) parameterized the renewal rate of the corresponding populations in the model. They were extracted from the literature and field observations (Table 1). All other host parameters were unchanged from the Cambodia model [36].

**Figure 1:**
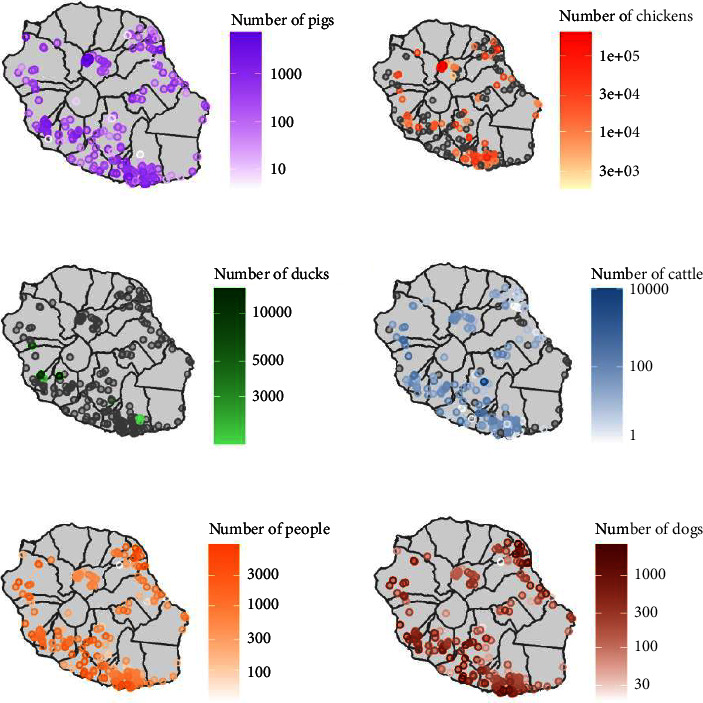
Host population sizes by epidemiological unit. Grey circles correspond to epidemiological units where the species of concern was absent.

In each epidemiological unit, population sizes of each host species (swine, chickens, ducks, cattle, dogs, and humans) were calculated (Figure 1) by intersecting a 1-km radius buffer zone drawn around the pig farm with GIS layers of either farm locations and sizes for production animals (a farm and its animals were counted as long as they were within the buffer zone) or population densities for humans and dogs (Figure 1).

Reunion Island pig farms were generally located at similar low and medium elevations, and because of the small size of the island, we assumed that environmental and climatic conditions that might affect vector abundance varied only slightly from farm to farm. For this reason and since *Cx. quinquefasciatus* larval sites were supposed to be related to the farming activities (e.g., manure pit) [40], we assumed that, at a given date, the size of the vector population did not vary between epidemiological units.

The available entomological data did not allow us to determine the real vector population size in the epidemiological units. In the Cambodian model, this size had been estimated at 48,663 vectors for an epidemiological unit (the village) of about the same size of our units here. In the present study, arbitrary values, although centered around the estimate in Cambodia to keep them realistic, of vector population sizes ranging from 100 to 100,000 vectors per unit were tested. By varying the size of the vector population in this systematic way, we explored the values that *R*_0_ would take according to different seasonal and other environmental conditions that influence vector populations dynamic. Other vector parameters were unchanged from the Cambodia model, including the feeding preference of *Cx. quinquefasciatus* for dogs relative to pigs, which had been calibrated from Cambodian field data [36].

### 2.4. *R*_0_ Calculation and Size of the Human Population Potentially Exposed to JEV

For a vector population size ranging from 100 to 100,000 vectors in steps of 1,000, the model estimated the value of *R*_0_ in each epidemiological unit using the next generation matrix method. The methodology is described in Ladreyt et al. [36]. Maps of epidemiological units with *R*_0_ greater or smaller than 1 were then created for 6 arbitrary vector population sizes (100, 500, 1,000, 10,000, 48,663—the estimated value in the Cambodian model, and 100,000 vectors).

We first aimed at calculating a potentially exposed human population size if JEV were introduced in a single epidemiological unit but considering situations where it could freely spread between adjacent units where *R*_0_ ≥ 1. For each value of the vector population size (from 100 to 100,000 vectors in steps of 1,000), we first filtered the epidemiological units where *R*_0_ was ≥1 and computed the adjacency matrix between these units based on the intersection between the 1 km radius buffers. This adjacency matrix allowed generating the corresponding network of neighborhood between units. The network —units being nodes— was then broken down into components (directly or indirectly linked nodes), which may contain only one node. Finally, the size of the human population exposed to JEV following its introduction in a given epidemiological unit was computed as follows:

- if *R*_0_ was <1 in this epidemiological unit, the size of the exposed population was 0;
- if *R*_0_ was ≥1, exposed people were people living in this unit or in those where JEV would be able to spread following neighborhood relationships. By construction, this corresponded to the component of the neighborhood network to which the unit belonged. We thus calculated the geographical area formed by the spatial union of the units included in this component (based on the associated 1 km buffers). The exposed population size was finally the number of people living in this geographical area (this value was therefore identical for all the epidemiological units of the same component).

We thus obtained 255 values of potentially exposed population size that formed a distribution from which we extracted the median and the 2.5% and 97.5% percentiles and plotted them against the vector population size.

In a second step, the sum of the inhabitants of all units with *R*_0_ ≥ 1 (i.e., the spatial union of the associated 1 km buffers) was plotted against the size of the vector population per unit.

As young people develop more severe forms in endemic areas [59], we also generated the abovementioned results specifically for individuals under 17 years old and plotted them on the same graph.

The model was developed and run on R software version 4.0.2 and results were computed using *sf* (1.0.7), *ggplot2* (3.3.5), *dplyr* (1.0.7), *tidyverse* (1.3.1), *sp* (1.4.6), *rgdal* (1.5.32), *maptools* (1.1.3), *rgeos* (0.5.9), *crop* (0.0.2), *raster* (3.5.15), *ggspatial* (1.1.5), and *igraph* (1.2.9) 4.1.1 R packages.

## 3. Results

### 3.1. *R*_0_ Estimation in Epidemiological Units

**Figure 2:**
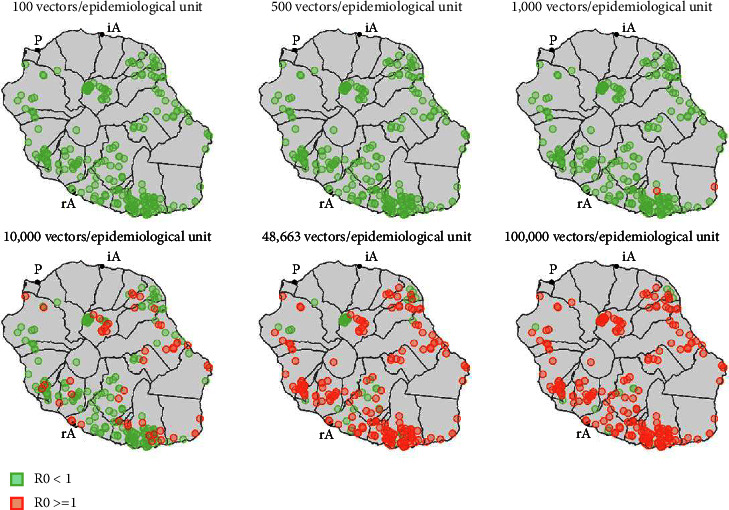
Epidemiological units classified according to their estimated *R*_0_ value, for 6 arbitrary vector population sizes. P: port area, iA: international airport, and rA: regional airport.

Figure 2 provides a visualization of the location of epidemiological units with *R*_0_ ≥ 1 for each size of the vector population. The proportion of epidemiological units where *R*_0_ was greater than 1 increased with the vector population size within the unit. If introduced, JEV could invade 75% of the identified epidemiological units in Reunion Island if the vector population size were similar to the one estimated by the model in Cambodia (48,663 vectors per epidemiological unit [36]). Two epidemiological units in the south of the island had an *R*_0_ ≥ 1 with only 1,000 vectors in the unit. The details of the compositions of these two units in terms of host population sizes and proportions provided in supplementary material (S1) show that the vector/hosts and competent hosts/noncompetent hosts ratios in these two units are high. However, the relation between the composition of the epidemiological units and *R*_0_ is complex and nonlinear, as shown in Ladreyt et al. [36], and was not studied further because not in the scope of this paper.

An epidemiological unit located in the neighborhood of the port area, a potential gateway for JEV (“P,” Figure 2), had an *R*_0_ ≥ 1 with 10,000 vectors and more. Similarly, several units where *R*_0_ ≥ 1 with 10,000 vectors and more were located near the regional airport in Saint-Pierre (“rA,” Figure 2). There were no epidemiological units near the international airport in Saint-Denis (“iA,” Figure 2).

### 3.2. Size of the Human Population Potentially Exposed to JEV

**Figure 3:**
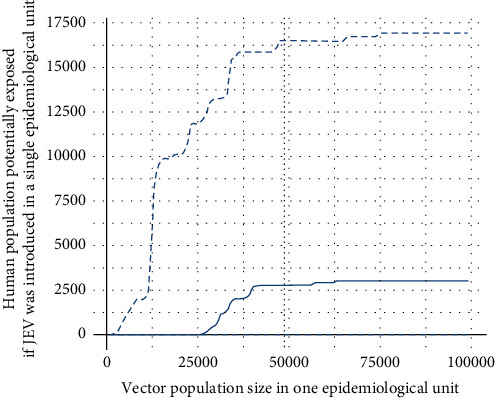
Size of the human population potentially exposed to JEV if the virus was introduced in a randomly selected epidemiological unit (*N* = 255), as a function of the size of the vector population per unit. The plain line represents the median, the upper-dashed line represents the 97.5% percentile, and the lower-dashed line represents the 2.5% percentile. The vertical dotted line represents the vector population size estimated by the model in Cambodia for a unit of similar size.

The first indicator represented the exposed population size in a situation where JEV would be introduced into a single epidemiological unit and circulate there as well as in the adjacent units where *R*_0_ ≥ 1. In this case, the median size of the human population potentially exposed to JEV would start increasing from 25,000 vectors per unit and would be capped at about 3,000 exposed people if the vector population per unit continued to increase (Figure 3). The 97.5% percentile of this distribution would reach about 16,500 potentially exposed people with about 50,000 vectors per unit, which would represent a little less than 2% of the Reunionese population (Figure 3).

**Figure 4:**
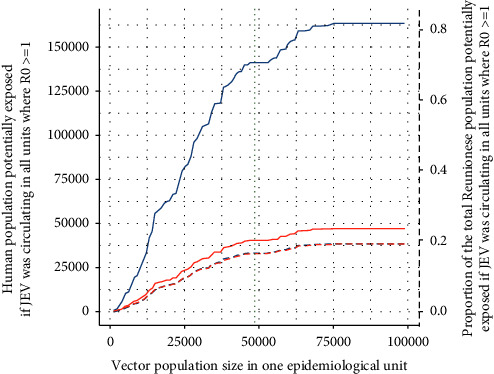
Number of people (solid lines) and proportion of the total Reunionese population (dashed lines) living in all units where the estimated *R*_0_ ≥ 1 as a function of the size of the vector population per unit. Blue lines: whole population and red lines: <17 years old population. The vertical dotted line represents the vector population size estimated by the model in Cambodia for a unit of similar size.

The second indicator represented the maximum exposed population size in a situation where JEV would have spread all over the island and would circulate in the epidemiological units where *R*_0_ ≥ 1. In that case, nearly 80,000 people (including 25,000 < 17 yrs old), would be potentially exposed to JEV with 25,000 vectors per unit. If the vector population size exceeded about 60,000 vectors per unit, up to about 20% of the Reunionese population, i.e. up to 175,000 people including slightly less than 50,000 < 17 yrs old, would live in epidemiological units where the estimated *R*_0_ was ≥1 (Figure 4).

## 4. Discussion

Ancient and contemporary history is replete with numerous examples of the spread of vectors and with them of diseases as technical progress was made in the field of maritime and air transport [5]. The introduction of JEV into a disease free region could indeed result from the introduction of infected vectors and/or infected competent hosts; although for Reunion Island, this second hypothesis seems less likely because the importation of live livestock (pigs and poultry) is prohibited [60]. Infected vectors could also be introduced by passive flight, carried by the wind (considered to be one of the causes of introduction of JEV to northern Australia from Papua New Guinea [61]), but given the distance between Southeast Asia and Reunion Island, this hypothesis seems less likely than a commercial introduction for Reunion Island, unless the virus was first introduced to Mauritius (about 200 km away) or Madagascar (about 800 km away). Numerous commercial lines exist between Reunion Island harbor, Japan, Hong Kong, India, and the whole of Southeast Asia and ensure the importation of goods from China, Thailand, and India by cargo ship in 18–30 days [46, 47], which is compatible with the survival of infected *Culex* spp. on board, living on average 25 days [48]. Indeed, an introduction of JEV from an endemic area by infected vectors would result primarily from an introduction of adult mosquitoes because, unlike dengue virus in *Aedes* spp., transovarial transmission of JEV in *Culex* spp. occurs only rarely [62].

In this study, we estimated *R*_0_ in epidemiological units composed of potential competent and noncompetent hosts in Reunion Island. In these units, host population sizes were fixed based on field data. Since vector population sizes were known to vary with season, we systematically explored populations sizes varying between 100 and 100,000 vectors. While the calculation of *R*_0_ using a deterministic model allowed us to quantify the ability of JEV to invade the epidemiological system present where it is introduced, our results should not be interpreted in terms of probabilities that this invasion will occur. Indeed, stochastic effects play an important role in the introduction of a pathogen into an uninfected population, which may result in early extinction even in an area where *R*_0_ ≥ 1. It would be necessary to use a stochastic model to quantify a local circulation probability of JEV in case of introduction. Our results show that, for sufficient vector population sizes, JEV may be able to invade part of these epidemiological units. Of particular interest are those located near potential gateways for the introduction of JEV into Reunion Island, i.e*.,* ports and airports, especially those connecting JEV endemic regions to the island. With a vector population size of 10,000 vectors/unit, which could happen since this corresponds to about 5 times fewer vectors estimated in Cambodia for a similar size unit [36], one epidemiological unit located within a few kilometers of the island's port area was at risk (*R*_0_ ≥ 1) (Figure 2). This represents a significant risk of JEV circulation in this unit if infected vectors were introduced by cargo. No pig farms were identified in the vicinity of the international airport, but several at-risk units were located in the vicinity of the regional airport (Figure 2), although the risk of introduction through this gateway is likely to be lower because this airport primarily operates short-distance regional flights. Besides these potential gateways, a more diffuse risk of JEV introduction throughout the island exists, linked to the import and transport of containers, whose final destinations are multiple. Some of the epidemiological units were at risk of being invaded by JEV in case of introduction even with relatively small vector population sizes (1,000 vectors/epidemiological unit). Almost 20% of the epidemiological units could be invaded by JEV with 10,000 vectors per unit. For comparison purposes, and all parameters and conditions considered equal, more than 75% of the units would have an estimated *R*_0_ ≥ 1 and around 140,000 inhabitants would be potentially exposed to JEV if the virus was introduced in every unit if the vector population size was similar to that estimated in Kandal region near Phnom Penh, Cambodia (48,663 vectors per unit [36]).

Beyond the size of the vector population, the importance of enzootic transmission depends on the trophic behavior of the mosquitoes for the different host species, the availability of these species and the capacity of the virus to replicate in these species. These parameters vary from one region to another within the same species (of host or vector) and can directly impact the dynamics of virus transmission through differences in vector competence [63] and feeding preferences [64] from one region to another.

The vector competence parameters values we used in the model came from experimental studies conducted in different regions [65–70]. To refine our analysis, it would be necessary to evaluate the competence for JEV of the Reunionese populations of *Cx. quinquefasciatus*, and that of *Cx. tritaeniorhynchus* and *Cx. neavei* (not present in JEV endemic regions) which has been shown to be a potential vector of other flaviviruses (WNV and USUV) [71, 72]. In addition, since European populations of *Ae. albopictus* have been experimentally shown to be competent for JEV [35], it would also be necessary to evaluate the competence of local populations of this species, which is very abundant all over the island. Secondly, experimental studies have shown that temperature can have an impact on vector competence for JEV [73–75]. The fact that pig farms in Reunion Island are located at similar altitudes suggests small temperature differences between farms during the same season. However, the temperature differences could be important within the same farm between summer and winter and could impact vector competence. Although vectors were shown to be competent for JEV in laboratory studies at both the lowest (18°C) and highest (34°C) average temperatures recorded in a year on the periphery of the island [50], they did not present the same transmission rates at these temperatures [73–75].

The values of the host-feeding preference parameters in this model came from a field study conducted in Kandal, Cambodia [39]. To refine our analysis, it would be relevant to investigate the feeding preferences of *Cx. quinquefasciatus* populations in Reunion Island, in the local eco-climatic context, as it has already been done for *Ae. albopictus * [76].

Additional data on vector competence, feeding preferences, and abundance of the different potential JEV vector species would also allow assessing the extent to which our simplifying assumption of considering only one vector population (*Cx. quinquefasciatus*) is valid, or whether it would be necessary to consider each *Culex* species separately (*Cx. tritaeniorhynchus*, and *Cx. quinquefasciatus*), or to include *Ae. albopictus* in the model.

Reunion Island has a steep landscape with a central peak reaching 3,074 meters. *Culex quinquefasciatus* is abundant in all coastal areas of the island but also in midaltitude areas up to 800 m and can be found up to 2,000 m in summer (Reunion Island regional health agency (ARS), pers com). As epidemiological units did not exceed 1,000 m in altitude, we did not take this altitude factor into account in the model. In addition to the fact that vector abundance is expected to decrease with altitude, environmental and climatic conditions are highly variable over very short distances on the island, and in the most studied mosquito species, i.e., *Ae. albopictus*, this leads to very strong spatial (west vs. east) variations in the density of breeding sites, even at low altitude [77]. This is not the case for *Cx. quinquefasciatus* which develops in large and rather stable breeding sites (collections of stable and permanent polluted water with the presence of organic matter). In particular, manure pits are always present in pig farms, and they are used yearlong. The constant availability of this breeding site justifies our assumption that *Cx. quinquefasciatus* abundance varies only slightly from one farm to another. The size of the farm could however influence this abundance, by having an impact on the number and size of the manure pits and thus on the size of the larval breeding sites, as well as on the number of different hosts from which to feed which enables egg development. More field data are needed to validate or invalidate our hypothesis of low spatial variations in *Cx. quinquefasciatus* population sizes in pig farms and to investigate the impact of farm size on vector population size.

Our study considered epidemiological units centered on pig farms because these are the main competent hosts and because we considered that each one hosted at least one *Cx. quinquefasciatus* breeding site. However, since circulation in domestic birds may allow JEV to occur in pig-free regions [36], units centered on poultry farms could be considered, although this requires evidence of the presence of vector breeding sites in the area. On the other hand, *Cx. triaeniorynchus* although rare in Reunion Island, has been found on the coast in natural areas surrounding ponds and wetlands [40], where some species of ardeids like the striated heron (*Butorides striata*) can live and nest [78]. Although discreet, this bird could act as a reservoir for JEV, but its potential as a reservoir for JEV needs to be investigated. The low abundances of *Cx. triateniorynchus* and heron nevertheless suggested that we should focus initially on domestic systems that harbor JEV-amplifying species and represent an increased risk to human health due to their proximity to areas of high human density.

According to our results, if JEV was introduced in a single epidemiological unit, the size of the potentially exposed human population would be relatively small, capping at a median of 3,000 people (and a 97.5% percentile of 16,500 people). In this case, the presence of JEV might not be detected until late because the proportion of clinical cases among exposed individuals is low, although possibly underestimated [79] and because JEV is currently neither part of the differential diagnosis of encephalitis or flavivirus infections in humans, nor of reproductive disorders in pigs (abortions and mortality of newborns) in Reunion Island. Once introduced, in addition to the risk of long circulation in the unit of introduction because of (i) the batch management of pig herds, which ensures a continuous renewal of susceptible competent hosts and (ii) the presence of potential JEV vectors all year round in the farms since manure pits are larvae breeding sites, the virus could spread more or less widely before being detected.

Model estimations suggest that, in the worst-case scenario where the virus would have spread all around the island and would circulates in all units where *R*_0_ ≥ 1, up to 20% of Reunion Island population would be exposed to JEV and thus at risk of being infected.

The probable delay between the introduction of JEV and the identification of the first clinical case might justify setting up JEV surveillance in Reunion Island. In early 2022, JEV was detected in mummified, stillborn, and weak newborn piglets from multiple commercial piggeries across eastern and southern Australia [80], with concomitant recognition of human cases of JE [81]. First, JE should be included in the differential diagnosis of human encephalitis in Reunion Island. Surveillance of reproductive failures and mortality in pig farms (especially those located near the port area), as well as awareness raising among veterinarians and farmers, could also be implemented. Second, a passive entomological surveillance system (e.g., using baited nucleic acid preservation card such as FTA™ cards) could also be considered at the potential entry points of JEV and especially in the port area, to detect the possible introduction of mosquito vectors infected with JEV [82, 83]. In addition, a routine serological surveillance of pigs at the slaughterhouse could be implemented to monitor a possible emergence of JEV on the island. Some species of ardeids are present in Reunion Island like the striated heron (*Butorides striata*) in majority although not very common, the cattle egret (*Bubulcus ibis*) and the Malagasy pond heron (*Ardeola idea*) which are both very rare [78]. Given the low densities, surveillance of these species may be considered but as a second line.

Finally, these measures should be associated with a reinforcement of the communication on the protection of people against mosquito bites, already in place to fight against culicidal nuisance and dengue epidemics, as well as communication to pig farmers and veterinarians.

## Figures and Tables

**Table 1:** Reunion Island specific model parameters.

Parameters	Definition	Value	References
1/*μ*_p_	Average lifespan of pigs	6.5 m	[45]
1/*μ*_d_	Average lifespan of ducks	85 d	[57]
1/*μ*_c_	Average lifespan of chickens	55.6 d	[57]
1/*μ*_b_	Average lifespan of cattle	5.7 y	[54, 55]
1/*μ*_dog_	Average lifespan of dogs	5.9 y	[56]
1/*μ*_h_	Average lifespan of humans	80.5 y	[58]

Subscripts in “Parameters” column: p = pigs, d = ducks, c = chickens, b = cattle, h = human; Subscripts in “Value” column: m = month, d = day, y = year.

## Data Availability

The data used to support the findings of this study are available from the corresponding author upon request.

## Supplementary Materials

Supplementary Materials S1: Compositions, in terms of host population sizes, and estimated *R*_0_ values of the 255 epidemiological units, for 1,000 vectors per unit. The ranking of units according to their *R*_0_ value (column A) was the same regardless of vector population size.
