# Supplementary material for: Modelling the Potential Human Exposure to Japanese Encephalitis Virus (JEV) in Case of Introduction into Reunion Island
Source: Transbound Emerg Dis. 2023 Jun 20;2023:3118640. doi: 10.1155/2023/3118640 (PMC12017079; doi:10.1155/2023/3118640)
Supplement: Supplementary Materials — S1: Compositions, in terms of host population sizes, and estimated R0 values of the 255 epidemiological units, for 1,000 vectors per unit. The ranking of units according to their R0 value (column A) was the same regardless of vector population size. [file 3118640.f1.pdf]

unit_ranking	pigs&sows	ducks	chickens	turkeys	guinea_fowls	cattle	humans	stray_dogs	domestic_dogs	vector_population	estimated_R0
1	20	0	0	0	0	0	86.86858981	29.94225678	22.86015521	1000	1.30532783720752
2	142	0	0	0	0	0	146.7866495	35.61075959	38.62806565	1000	1.07682521773667
3	80	0	0	0	0	5	175.3686185	1.450737987	46.14963644	1000	0.996137466211214
4	408	0	0	0	0	0	69.96960715	26.72160293	18.41305451	1000	0.913673504826963
5	325	0	0	0	0	0	171.2008031	20.44615325	45.05284292	1000	0.873588841378544
6	180	0	0	0	0	8	217.8171235	22.04050648	57.32029565	1000	0.871060144337048
7	528	0	0	0	0	0	37.07530045	24.28944061	9.756658013	1000	0.846432941016076
8	649	0	0	0	0	0	33.65076801	9.884904573	8.855465266	1000	0.773628089284026
9	474	0	0	0	0	2	250.7626651	2.853592318	65.99017503	1000	0.722905371219575
10	254	0	0	0	0	37	381.1932873	45.76370945	100.314023	1000	0.615428091742377
11	465	0	0	0	0	0	458.336722	45.53252039	120.6149268	1000	0.612231640083249
12	465	0	0	0	0	0	459.6830506	45.45953186	120.9692238	1000	0.611625774657565
13	533	0	0	0	0	9	423.7877113	141.6112561	111.5230819	1000	0.591647876472742
14	490	0	0	0	0	10	478.0343475	26.72160293	125.7985125	1000	0.587344626274717
15	309	0	0	0	0	0	573.285393	46.60224466	150.8645771	1000	0.575519634952161
16	533	0	0	0	0	12	526.2687388	83.52555017	138.4917734	1000	0.554858490823865
17	41	0	0	0	0	0	345.7630852	115.9924119	90.99028558	1000	0.543141032997134
18	490	0	0	0	0	28	557.5652998	26.72160293	146.7277105	1000	0.536200885095041
19	1414	0	0	0	0	0	109.2414234	6.585237109	28.74774299	1000	0.518315027524448
20	288	0	0	0	0	129	299.8646241	67.53326575	78.91174319	1000	0.509876386247912
21	288	0	0	0	0	129	304.5377185	67.53326575	80.14150487	1000	0.508004259235118
22	196	0	0	0	0	16	629.3462643	8.756753239	165.617438	1000	0.50796569412487
23	711	0	0	0	0	29	591.0884347	126.6385328	155.5495881	1000	0.495846780562801
24	473	0	0	0	0	7	755.8912389	77.70261796	198.9187471	1000	0.491979167954339
25	583	0	0	0	0	17	731.6517721	175.2447242	192.53994	1000	0.478835361602307
26	245	0	0	0	0	0	817.8061542	36.35926042	215.2121458	1000	0.463599193824151
27	478	0	0	0	0	85	654.8180296	19.06164899	172.3205341	1000	0.4557718346788
28	517	0	0	0	0	60	751.1983341	16.73278496	197.6837721	1000	0.451863628787324
29	929	0	0	0	0	0	831.6315438	0	218.8504063	1000	0.450843880473714
30	1414	0	0	0	0	101	295.1708411	10.75663902	77.67653713	1000	0.437712437544703
31	711	0	0	0	0	29	931.6953612	148.6382187	245.1829898	1000	0.424232880926085

32	886	0	0	0	0	4	1133.422674	54.38748093	298.2691249	1000	0.401763829363936
33	2383	0	0	0	0	0	197.4894482	32.39000896	51.97090742	1000	0.397831429984152
34	179	0	0	0	0	48	776.8011474	100.9758319	204.4213546	1000	0.395411072391365
35	487	0	0	0	0	35	1117.822074	17.15601535	294.1637037	1000	0.391415104742874
36	309	0	0	0	0	0	1155.956376	46.12327313	304.1990462	1000	0.383588323088649
37	629	0	0	0	0	1	1295.303168	282.3528924	340.8692547	1000	0.375501642697689
38	295	0	3500	0	0	16	704.9205115	159.8527602	185.5053978	1000	0.366782471916862
39	295	0	3500	0	0	45	616.7842648	169.0497457	162.3116486	1000	0.365544284787253
40	308	0	6900	0	0	44	234.1877646	0	61.62835911	1000	0.365468064679526
41	182	0	0	0	0	114	683.6822304	75.02952422	179.9163764	1000	0.36543207720239
42	392	0	3708.75	0	0	36	718.6760589	94.27780431	189.1252787	1000	0.363690690339224
43	242	0	0	0	0	41	1050.603893	7.121571338	276.4747088	1000	0.362003454707968
44	242	0	0	0	0	19	1141.713286	14.04656972	300.4508648	1000	0.357096508136958
45	196	0	0	0	0	7	1156.216899	22.16411499	304.2676049	1000	0.343657211087241
46	526	0	9000	0	0	0	457.6386569	56.69007231	120.4312255	1000	0.329278836597433
47	526	0	9000	0	0	0	457.6386569	56.69007231	120.4312255	1000	0.329278836597433
48	2218	0	0	0	0	10	1251.907121	229.8869573	329.4492423	1000	0.32614095673997
49	1321	0	1800	0	0	97	1199.482575	203.604644	315.6533091	1000	0.322334053437008
50	569	0	0	0	0	72	1547.122675	110.4817072	407.1375461	1000	0.316607369180442
51	796	0	0	0	0	28	1857.717511	80.31939764	488.8730293	1000	0.310642180279293
52	921	0	0	9608.75	0	35	540.6928116	17.15601535	142.287582	1000	0.310338067792975
53	18	0	0	0	0	3	477.3629266	40.77302439	125.6218228	1000	0.305514065927914
54	1467	0	0	0	0	2	2021.175065	52.74392041	531.8881749	1000	0.304357989669319
55	1184	0	0	0	0	377	929.6849768	0	244.6539413	1000	0.300968573585685
56	1184	0	0	0	0	377	929.6849768	0	244.6539413	1000	0.300968573585685
57	1184	0	0	0	0	377	929.6849768	0	244.6539413	1000	0.300968573585685
58	1184	0	0	0	0	377	929.6849768	0	244.6539413	1000	0.300968573585685
59	468	0	11300	0	0	1	389.2895375	124.5204852	102.4446151	1000	0.300296795299057
60	39	0	0	0	0	0	711.4600259	80.20486282	187.2263226	1000	0.298115561149046
61	188	0	0	0	0	82	1152.464178	21.69755161	303.2800469	1000	0.294846273991163
62	921	0	0	9608.75	0	104	569.0813831	17.15601535	149.7582587	1000	0.292217090716759

63	748	0	10725	0	0	71	482.0152643	49.41815936	126.8461222	1000	0.292108620706114
64	748	0	10725	0	0	71	482.0152643	49.41815936	126.8461222	1000	0.292108620706114
65	748	0	10725	0	0	71	482.0152643	49.41815936	126.8461222	1000	0.292108620706114
66	748	0	10725	0	0	71	482.0152643	49.41815936	126.8461222	1000	0.292108620706114
67	9	0	0	0	0	30	280.9434124	0	73.93247694	1000	0.290670943039144
68	491	0	0	0	0	18	1902.075731	127.0663409	500.5462449	1000	0.290000606251608
69	1660	0	0	0	5362.5	64	1341.85993	15.46240045	353.1210343	1000	0.285321846885891
70	780	0	10725	0	0	69	584.6507086	91.44912961	153.8554496	1000	0.285225671101226
71	1201	0	0	0	8550	47	1053.911172	31.0487018	277.3450452	1000	0.28309582499859
72	394	0	0	0	0	0	1886.850432	202.4541601	496.5395874	1000	0.282858899626854
73	950	0	7875	0	0	127	915.1783858	45.8297145	240.8364173	1000	0.280304251397634
74	886	0	0	0	0	3	2371.79748	67.1414407	624.1572316	1000	0.279255384536012
75	1813	0	0	0	0	2	2434.766328	45.64153418	640.7279812	1000	0.277073424446663
76	1813	0	0	0	0	2	2434.766328	45.64153418	640.7279812	1000	0.277073424446663
77	1813	0	0	0	0	2	2434.766328	45.64153418	640.7279812	1000	0.277073424446663
78	1813	0	0	0	0	2	2434.766328	45.64153418	640.7279812	1000	0.277073424446663
79	1321	0	5430	0	0	97	1436.792384	202.1696689	378.1032589	1000	0.274995244700913
80	1679	0	0	0	0	79	2290.879585	95.04229449	602.8630488	1000	0.273058050119089
81	251	0	0	0	0	92	1447.373531	46.72763269	380.8877713	1000	0.271542620512128
82	1813	0	0	0	0	0	2641.122606	32.74696966	695.0322648	1000	0.268056670138721
83	1321	0	5430	0	0	118	1512.020244	224.607595	397.9000642	1000	0.267398726205204
84	1321	0	5430	0	0	118	1512.020244	224.607595	397.9000642	1000	0.267398726205204
85	1321	0	5430	0	0	118	1512.020244	224.607595	397.9000642	1000	0.267398726205204
86	629	0	0	0	0	1	2345.853003	338.2833391	617.3297376	1000	0.264833778426995
87	526	0	9000	0	0	0	1203.548473	69.36043923	316.7232823	1000	0.263136536429721
88	526	0	9000	0	0	0	1203.548473	69.36043923	316.7232823	1000	0.263136536429721
89	830	0	0	0	0	6	2592.490398	38.27771635	682.2343153	1000	0.262705560059886
90	843	0	0	0	0	0	2645.364142	43.54027236	696.1484584	1000	0.261046000251113
91	830	0	0	0	0	6	2627.9874	39.04599696	691.5756316	1000	0.260481795086578
92	773	0	10500	0	0	123	850.2494731	29.93396033	223.7498614	1000	0.259904702396756
93	758	0	0	0	0	58	2395.10126	170.8218049	630.2898053	1000	0.259497654089624
94	300	0	0	0	0	225	1241.532653	88.17644536	326.7191193	1000	0.259278872557236
95	1201	0	0	0	8550	47	1548.72752	36.3202507	407.5598736	1000	0.257123734379158

96	1813	0	0	0	0	2	2905.687598	37.40214838	764.6546312	1000	0.256527349432891
97	830	0	0	0	0	39	2646.150756	41.72634441	696.3554621	1000	0.253030215697309
98	2094	0	9750	0	0	13	1445.798083	278.4837019	380.4731798	1000	0.252961663065412
99	773	0	12140	0	0	123	771.1349747	22.31541268	202.9302565	1000	0.252297340008528
100	773	0	10500	0	0	123	1039.605619	28.81082387	273.580426	1000	0.249368390992331
101	1660	0	0	5750	5362.5	64	1378.18485	11.9440409	362.6802236	1000	0.24764279031687
102	519	0	2000	5750	0	80	1389.895646	12.41790257	365.7620122	1000	0.244543363672
103	783	0	0	0	0	390	1705.097751	5.860060715	448.7099344	1000	0.241264750007577
104	1436	1800	9750	0	0	13	1566.411383	251.39026	412.2135217	1000	0.240722807575928
105	1168	1500	12100	0	2000	128	794.6204344	17.68296756	209.1106406	1000	0.238456610976649
106	1201	0	0	0	8550	66	1943.552212	33.78753261	511.4611083	1000	0.237170585895284
107	2094	1800	9750	0	0	30	1555.811232	273.2580427	409.4240083	1000	0.233787706856781
108	569	0	0	0	0	81	2591.128753	119.256262	681.8759876	1000	0.23036695465246
109	1215	0	14590	0	0	42	1598.353514	43.03123034	420.6193459	1000	0.222610898257811
110	906	0	15050	0	0	147	1106.496029	70.67095177	291.1831655	1000	0.219905755890251
111	2393	0	14590	0	0	93	1617.205288	27.98417694	425.580339	1000	0.219468125071335
112	1669	0	0	8000	8000	108	1446.136768	41.79586879	380.5623074	1000	0.218147071317357
113	1189	0	18000	0	0	82	1145.647388	15.00955061	301.4861549	1000	0.218003971374967
114	591	0	20800	0	0	11	672.9493702	56.3427675	177.0919395	1000	0.214687544018012
115	777	0	7875	0	0	128	2153.391352	45.48152219	566.6819346	1000	0.212779849695422
116	758	0	0	0	0	76	3236.539538	184.1156359	851.7209311	1000	0.212165656609662
117	359	0	0	0	0	197	2100.318186	17.15601535	552.7153121	1000	0.211887915806089
118	869	0	18200	0	0	73	1164.749845	59.21428849	306.513117	1000	0.210819388541817
119	869	0	18200	0	0	73	1164.749845	59.21428849	306.513117	1000	0.210819388541817
120	294	0	11500	0	0	0	1417.191827	37.24075807	372.9452177	1000	0.210548628847671
121	260	0	19075	0	0	0	541.3249123	6.79721042	142.4539243	1000	0.21004731985267
122	627	0	0	0	0	85	3160.256741	41.8450652	831.6465108	1000	0.206561845727974
123	1202	0	22100	0	0	41	1153.278528	10.4059622	303.4943496	1000	0.205294566472398
124	391	0	0	0	0	441	1536.03967	44.3993761	404.2209658	1000	0.204348530491273
125	1669	0	0	8000	12840	108	1313.999153	29.75815845	345.7892508	1000	0.204060981112077
126	631	0	18640	0	0	34	1207.367466	12.06427605	317.7282805	1000	0.203818696473215
127	1100	7440	18736.875	0	0	172	95.41822974	40.58644415	25.11006046	1000	0.203313995353201
128	1201	0	8347.5	0	8550	50	1886.338756	54.54872067	496.4049359	1000	0.202964204109167

129	203	14400	0	0	0	29	1705.202998	97.43259469	448.737631	1000	0.200868660025358
130	1548	0	0	8000	12840	152	1283.521652	0	337.7688558	1000	0.200749396627392
131	203	14400	0	0	0	29	1806.621652	104.3611523	475.4267505	1000	0.199386525590765
132	580	0	23800	0	0	3	774.8455971	111.8178317	203.9067361	1000	0.19750065404708
133	772	0	19050	3800	0	113	795.2367059	16.8271787	209.2728173	1000	0.197006105943537
134	13	0	0	0	0	36	554.5853879	34.99607627	145.9435231	1000	0.196195136205984
135	657	0	0	8000	12840	105	1078.59677	0	283.8412553	1000	0.192757471888257
136	612	0	0	0	0	1	3750.017383	0.108962198	986.8466796	1000	0.192322763630926
137	2105	0	0	0	0	11	5078.586429	180.7643656	1336.470113	1000	0.191962518806132
138	1159	1500	26100	0	2000	94	804.0024116	12.23638785	211.579582	1000	0.189818555110271
139	2105	0	0	0	0	11	5191.272416	213.0920491	1366.12432	1000	0.189461969676298
140	239	0	0	0	0	7	2574.494169	288.3536968	677.4984656	1000	0.189280863333773
141	927	1500	26100	0	2000	94	758.8215611	12.09482685	199.6898845	1000	0.188965127450093
142	1159	1500	26100	0	2000	94	857.7055979	13.20959012	225.7119994	1000	0.188717772141327
143	1159	1500	26100	0	2000	94	857.7055979	13.20959012	225.7119994	1000	0.188717772141327
144	785	0	14590	0	0	2	2451.699082	102.9229082	645.1839688	1000	0.188322153492225
145	1360	0	16110	5750	5362.5	63	1172.844834	27.1629051	308.6433774	1000	0.188067357288684
146	412	0	22040	0	0	83	785.8572061	54.40542406	206.8045279	1000	0.187307875934258
147	294	0	11500	0	0	2	1955.179426	37.9918284	514.5209016	1000	0.184874069161988
148	20	0	0	0	0	34	749.746296	170.5072186	197.3016568	1000	0.183328296960178
149	376	0	0	0	0	41	3139.047812	299.4522561	826.0652138	1000	0.18262087224884
150	709	0	0	0	0	98	3915.499075	130.4448432	1030.394493	1000	0.181477919713272
151	20	0	0	0	0	185	314.7920165	41.78163729	82.84000434	1000	0.180045909851944
152	391	0	0	0	0	557	1670.520227	49.14156878	439.6105861	1000	0.179701741464129
153	22	0	0	0	0	0	932.3997557	94.94156013	245.3683568	1000	0.179574173634214
154	412	0	22040	0	0	86	1028.32872	50.52229945	270.6128209	1000	0.179214630977484
155	299	1500	26100	0	2000	90	757.0796347	24.11788841	199.2314828	1000	0.178676995368157
156	299	1500	26100	0	2000	90	757.0796347	24.11788841	199.2314828	1000	0.178676995368157
157	1100	7440	29236.875	0	0	173	254.1583153	43.09220496	66.88376717	1000	0.178298387484103
158	1791	0	34880	0	0	35	1068.956536	50.22322696	281.3043516	1000	0.175711430419087
159	6	0	11900	0	0	25	867.2180325	6.379611313	228.2152717	1000	0.17542128893817
160	2343	0	34880	0	0	40	1291.655106	23.11023428	339.9092384	1000	0.174311904745435
161	253	0	18200	0	0	2	1372.649031	547.947368	361.2234292	1000	0.173429551235825

162	1110	0	12400	0	0	148	3257.847546	41.05770107	857.3283016	1000	0.173373970641284
163	1791	0	34880	0	0	40	1248.590928	23.56901457	328.5765599	1000	0.172583295336738
164	313	0	24675	3300	0	60	497.1758773	1.465094865	130.8357572	1000	0.172414415027844
165	1202	0	22100	0	0	45	2520.364142	73.8140041	663.2537216	1000	0.172281080032746
166	323	0	0	0	0	405	2095.060939	20.67417675	551.331826	1000	0.171457904521155
167	1202	0	22100	0	0	44	2614.125251	61.15712805	687.9276976	1000	0.170557793430623
168	545	0	18800	0	0	392	998.1263098	0	262.6648184	1000	0.170302303878201
169	15	0	0	0	0	2	835.5829558	0	219.8902515	1000	0.169715807285047
170	1025	0	28690	3800	0	156	852.0025512	13.30102101	224.2111977	1000	0.16936717124171
171	142	0	0	0	0	31	2308.358736	54.42509973	607.4628253	1000	0.169111957128335
172	1344	0	12400	0	0	148	3785.733516	40.22217101	996.2456622	1000	0.16784242928144
173	153	0	9750	0	0	3	2069.881929	134.6082551	544.7057707	1000	0.163545891539117
174	227	0	0	0	0	0	3046.643745	168.237662	801.7483539	1000	0.163480428537447
175	702	0	0	0	0	2	4851.038483	186.177578	1276.589074	1000	0.162369236032118
176	1158	9300	26800	0	0	379	971.2504152	93.91683372	255.5922145	1000	0.162002047357861
177	385	0	16110	5750	5362.5	74	1094.681456	26.40196911	288.0740674	1000	0.161745325130442
178	1816	9300	26800	0	0	261	1420.853774	136.3215648	373.9088878	1000	0.159754310793268
179	391	0	0	0	0	539	2255.670953	41.71438465	593.5976192	1000	0.15967626516193 5
180	580	0	35800	0	0	77	730.6253726	116.2504309	192.2698349	1000	0.156930409640848
181	128	0	33000	0	0	14	183.9853252	219.01978	48.41719084	1000	0.156868268793467
182	832	0	27750	0	0	5	2346.743265	131.3174839	617.5640171	1000	0.156093409000811
183	261	0	0	0	0	87	3190.968203	25.96888681	839.7284745	1000	0.155235687627063
184	605	0	9460	0	0	98	3644.774262	120.3606995	959.1511215	1000	0.155140171264581
185	573	0	31700	0	0	9	1511.624045	303.7698372	397.7958012	1000	0.153863695819233
186	313	0	33975	3300	0	56	290.9443952	0	76.56431453	1000	0.153615649109929
187	772	0	37050	3800	0	113	787.1676457	16.53013351	207.1493804	1000	0.149633216931917
188	282	0	42210	0	0	0	95.65715462	0	25.17293543	1000	0.149296284304142
189	292	0	0	0	0	8	3751.983425	186.8395247	987.3640592	1000	0.149100142372938
190	2388	0	49470	0	0	42	1455.534651	26.67735285	383.0354344	1000	0.14790041869416
191	503	6000	50640	1500	0	171	85.70528005	27.05225444	22.55402106	1000	0.146462719984995
192	4	0	28530	0	0	32	509.0409915	46.778453	133.9581556	1000	0.14629096193196
193	991	0	12400	0	0	148	4540.412725	37.62210563	1194.845454	1000	0.145754416428903
194	503	0	47040	0	0	171	103.2454944	22.62350852	27.16986696	1000	0.139333470703208

195	503	0	47040	0	0	171	103.2454944	22.62350852	27.16986696	1000	0.139333470703208
196	442	0	24620	0	8220	83	1870.339945	179.334745	492.1947223	1000	0.13665587504355
197	442	0	27245	0	8220	193	1384.432522	126.208844	364.3243479	1000	0.134851350534453
198	407	0	0	0	0	94	4708.955268	16.45109267	1239.198755	1000	0.132962635288601
199	925	0	27750	0	0	3	4084.664918	128.7634623	1074.91182	1000	0.132570341649701
200	260	0	0	0	0	96	3928.830403	149.4042092	1033.902738	1000	0.129026645525632
201	486	0	50000	0	0	9	1050.454017	9.385204559	276.4352676	1000	0.128809714027153
202	600	0	0	0	0	5	6237.665158	6.695336939	1641.490831	1000	0.126425437836768
203	10	0	0	0	0	0	925.3662436	44.2264503	243.5174325	1000	0.126423763664974
204	1322	0	64800	0	0	10	1345.320963	61.9604696	354.0318324	1000	0.12347687995188
205	45	0	15800	0	0	69	2330.861153	41.39826871	613.384514	1000	0.121213340304534
206	7	0	0	0	0	68	614.9311884	0	161.823997	1000	0.120057082256599
207	5071	0	66745.75	25387	4800	43	370.9444763	0	97.61696746	1000	0.116752447591591
208	869	0	64800	0	0	3	1539.482751	58.46946207	405.1270397	1000	0.116019958172013
209	1962	0	84434.25	0	0	99	484.3056608	0	127.4488581	1000	0.115762737839007
210	4971	0	72695.75	22603	4800	35	362.5250218	0	95.40132153	1000	0.11492627985211
211	5895	0	76995.75	16587	17200	53	388.3410371	0	102.1950098	1000	0.109336696354731
212	2651	0	85640.25	4205	15400	81	560.1375023	0	147.4046059	1000	0.105357191173567
213	2335	0	85640.25	2784	15400	66	496.1124025	0	130.5558954	1000	0.105307849954845
214	5932	0	83195.75	16587	20200	53	376.0888879	0	98.97075997	1000	0.105025967030606
215	5071	0	76195.75	25387	20200	41	380.4496249	0	100.1183223	1000	0.103590987513489
216	6059	0	87974	16587	20200	53	457.9452744	0	120.5119143	1000	0.102743463158506
217	2125	0	106059.25	0	0	97	473.2980127	0	124.5521086	1000	0.102645742081302
218	4128	0	116737	2784	0	107	582.0248248	0	153.1644276	1000	0.101768725094324
219	5802	0	83195.75	25387	20200	43	411.2881445	0	108.2337222	1000	0.10111037620197
220	5802	0	83195.75	25387	20200	43	411.2881445	0	108.2337222	1000	0.10111037620197
221	6186	0	83195.75	25387	20200	61	447.8451971	0	117.8539992	1000	0.101099297985792
222	6186	0	83195.75	25387	20200	61	447.9807763	0	117.889678	1000	0.101098895059274
223	5932	0	83195.75	25387	20200	61	416.563501	0	109.621974	1000	0.101018748321506
224	6059	0	87974	25387	20200	53	433.1256975	0	113.9804467	1000	0.0992188010772011
225	5697	0	112426.75	17413	3000	71	386.4806301	0	101.705429	1000	0.0991780258391573

226	6186	0	87974	25387	20200	61	469.0418532	0	123.4320666	1000	0.0991345048549661
227	6186	0	87974	25387	20200	61	469.0418532	0	123.4320666	1000	0.0991345048549661
228	6839	0	108724	16587	17200	54	489.8225187	0	128.9006628	1000	0.0962232075224012
229	77	0	52080	0	0	17	2703.761734	38.29137192	711.5162459	1000	0.0959660496727243
230	132	0	0	0	0	21	4332.673697	172.2528213	1140.177289	1000	0.0934309610526628
231	6557	0	114924	16587	20200	53	503.7563677	0	132.5674652	1000	0.0929044798757255
232	6186	0	121926.75	25387	4800	81	444.6624323	0	117.0164296	1000	0.0924039886113418
233	6186	0	121926.75	25387	4800	81	444.6624323	0	117.0164296	1000	0.0924039886113418
234	6361	0	109324	25387	20200	59	482.4378104	0	126.9573185	1000	0.0917241827910347
235	45	0	64647	2900	0	4	2164.676334	189.5984702	569.6516667	1000	0.0909683917162255
236	6361	0	114924	25387	20200	59	493.7650583	0	129.9381732	1000	0.0899504928536849
237	6361	0	114924	25387	20200	59	502.985469	0	132.3645971	1000	0.0899296316508025
238	6361	0	114924	25387	20200	59	502.985469	0	132.3645971	1000	0.0899296316508025
239	4565	0	144155	15992	3000	85	327.8151433	0	86.26714296	1000	0.0870701440429182
240	6186	0	130205	25387	20200	81	483.3004176	0	127.1843204	1000	0.0852798452271337
241	7412	0	164055	16587	20200	107	614.0436562	0	161.5904358	1000	0.0798694717855742

242	7262	0	164055	16587	20200	95	592.3234894	0	155.8746025	1000	0.0798549805102136
243	7110	0	164055	16587	20200	101	619.1818755	0	162.9425988	1000	0.0796548391464798
244	6535	0	164055	14847	20200	93	581.6369272	0	153.0623493	1000	0.0796348839571484
245	6603	0	157155	25387	20200	94	536.8690844	0	141.281338	1000	0.0789711125746757
246	6361	0	157155	25387	20200	79	502.0463059	0	132.1174489	1000	0.0788706113740727
247	6361	0	157155	25387	20200	81	501.0712562	0	131.8608569	1000	0.078861527841378
248	292	0	0	0	0	449	6249.530133	318.018486	1644.613193	1000	0.0784421369983044
249	80	0	0	0	0	193	4538.641395	15.56200794	1194.379315	1000	0.0641515177077549
250	98	0	0	0	0	0	5716.107058	117.2288619	1504.238699	1000	0.0636782914481649
251	65	0	25040	0	0	5	9197.315108	279.2619325	2420.346081	1000	0.0590766400123486
252	38	0	0	0	0	47	3840.256721	200.627536	1010.593874	1000	0.057216731635315
253	5	0	0	0	0	52	2177.67833	121.7156102	573.0732448	1000	0.0358030646633421
254	263	0	7900	0	0	10402	1876.76752	17.69669089	493.8861895	1000	0.0202060739991319
255	263	0	7900	0	0	10402	2043.19385	19.74992235	537.6825921	1000	0.020115116856238
